# A fatal case of massive hemobilia caused by invasive pancreatic cancer with median arcuate ligament syndrome: A case report

**DOI:** 10.1097/MD.0000000000035701

**Published:** 2023-11-03

**Authors:** Ryosuke Nakatsubo, Atsushi Sofuni, Takayoshi Tsuchiya, Kentaro Ishii, Reina Tanaka, Ryosuke Tonozuka, Shuntaro Mukai, Kazumasa Nagai, Yukitoshi Matsunami, Kenjiro Yamamoto, Hiroyuki Kojima, Hirohito Minami, Noriyuki Hirakawa, Kyoko Asano, Takao Itoi

**Affiliations:** a Department of Gastroenterology and Hepatology, Tokyo Medical University, Tokyo, Japan

**Keywords:** hemobilia, median arcuate ligament syndrome, pancreatic cancer

## Abstract

**Introduction:**

In median arcuate ligament syndrome (MALS), the celiac artery is compressed, causing an arcade to develop in the pancreatic head, leading to ischemic symptoms and aneurysms.

**Patient concerns:**

The patient was diagnosed with borderline resectable pancreatic cancer (PC) and MALS. Endoscopic biliary drainage with a covered metal stent (CMS) was performed for the obstructive jaundice. After the jaundice improved, a modified FOLFIRINOX regimen was initiated. Several days later, cardiopulmonary arrest occurred after hematemesis occurred. Cardiopulmonary resuscitation was performed, his blood pressure stabilized, and emergent upper endoscopy was performed. The CMS was dislodged and active bleeding was observed in the papillae. The CMS was replaced, and temporary hemostasis was achieved. Contrast-enhanced computed tomography revealed a diagnosis of extravasation from the posterior superior pancreaticoduodenal artery (PSPDA) into the biliary tract. Transcatheter arterial embolization was performed. However, the patient was subsequently diagnosed with hypoxic encephalopathy and died on day 14 of hospitalization.

**Diagnosis:**

Biliary hemorrhage due to invasion of pancreatic cancer from the PSPDA associated with MALS.

**Intervention:**

None.

**Outcomes:**

Biliary hemorrhage from the PSPDA was fatal in the patient with invasive PC with MALS.

**Lessons:**

Since MALS associated with PC is not a rare disease, the purpose of this study was to keep in mind the possibility of fatal biliary hemorrhage.

## 1. Introduction

In median arcuate ligament syndrome (MALS), the celiac artery is compressed, causing an arcade in the pancreatic head and leading to ischemic symptoms and aneurysms. We report a case of biliary hemorrhage due to invasion of pancreatic cancer from the posterior superior pancreaticoduodenal artery (PSPDA) associated with MALS, which is fatal and should be considered.

## 2. Case presentation

**Figure 1.**
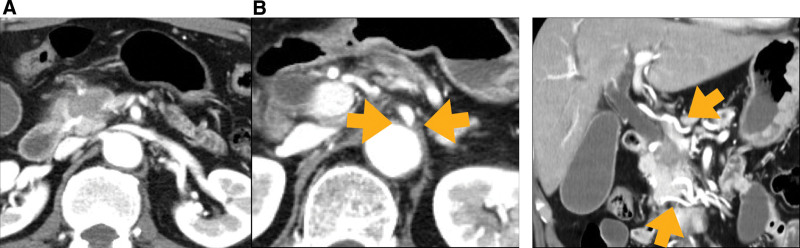
Contrast-enhanced CT scan showing (a) a hypovolemic mass in the pancreatic head and (b) stenosis of the origin of the celiac artery and development of a branch from the SMA (arrows). CT = computed tomography, SMA = superior mesenteric artery.

A 70-year-old man was referred to our department for treatment of obstructive jaundice. Blood tests showed elevated hepatobiliary enzymes and carbohydrate antigen 19-9 level. Contrast-enhanced computed tomography (CT) revealed a 3-cm mass in the pancreatic head with portal vein invasion (Fig. 1a). CT also showed stenosis in the celiac artery and prominent dilation of the arcade from the superior mesenteric artery (Fig. 1b). Adenocarcinoma was confirmed by endoscopic ultrasound-guided fine-needle biopsy. The diagnoses were borderline resectable PC (T2N0M0 stage IB) and MALS. Therefore, neoadjuvant chemotherapy was initiated.

**Figure 2.**
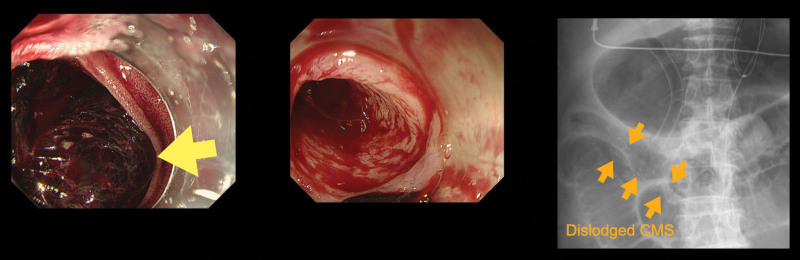
Upper gastrointestinal endoscopic image showing dislodgement of a CMS and active bleeding from the common bile duct. CMS = covered metal stent.

**Figure 3.**
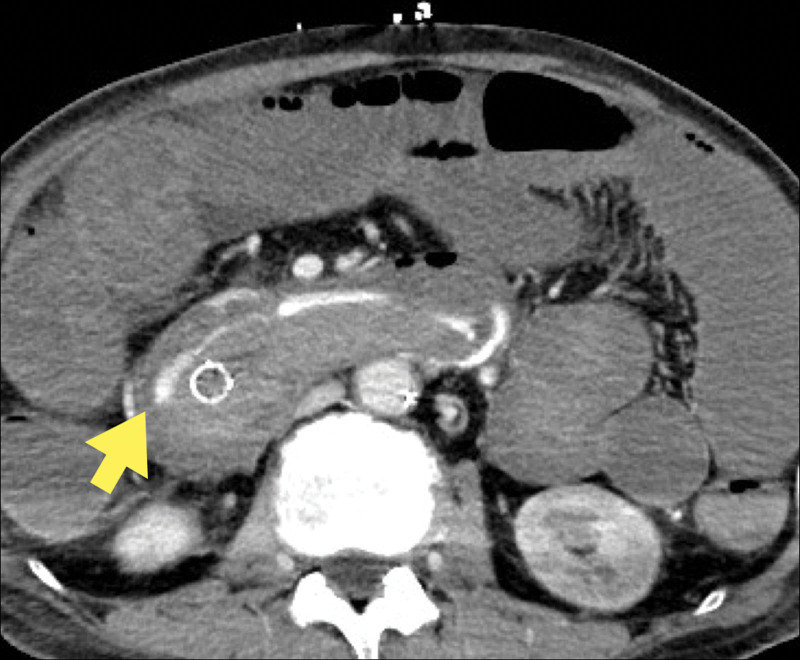
Contrast-enhanced CT scan showing extravasation from the PSPDA into the biliary tract. CT = computed tomography, PSPDA = posterior superior pancreaticoduodenal artery.

**Figure 4.**
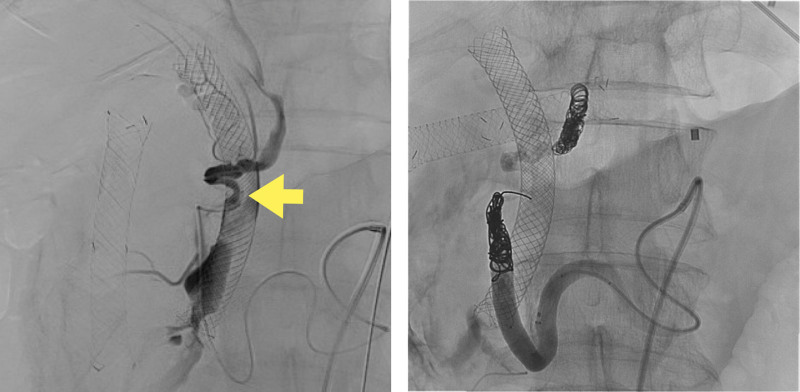
Angiogram of the PSPDA showing extravasation from the common bile duct to the duodenum (arrow). Extravasation disappeared upon TAE using coils and n-butyl cyanoacrylate. PSPDA = posterior superior pancreaticoduodenal artery, TAE = transcatheter arterial embolization.

Endoscopic biliary drainage with a covered metal stent (CMS) was performed for the obstructive jaundice. After the jaundice improved, a modified FOLFIRINOX regimen was initiated. Seven days after the administration of chemotherapy, the patient was admitted with a diagnosis of paralytic ileus caused by irinotecan. He improved with conservative treatment, but cardiopulmonary arrest after hematemesis occurred on hospital day 5. Cardiopulmonary resuscitation was performed, and the patient’s heart rate returned; however, but his blood pressure did not improve sufficiently. Resuscitative endovascular balloon occlusion of the aorta was performed. Once his blood pressure stabilized, an emergent upper endoscopy was performed. The CMS was dislodged, and active bleeding was observed in the papillae (Fig. 2). Therefore, the CMS was replaced and temporary hemostasis was achieved. Contrast-enhanced CT showed extravasation from the PSPDA to the biliary tract, and emergency transcatheter arterial embolization (TAE) was performed (Fig. 3). Abdominal angiography revealed disruption of the PSPDA due to tumor invasion, and hemostasis was achieved via TAE using coils and n-butyl-2-cyanoacrylate (Fig. 4). However, the patient was subsequently diagnosed with hypoxic encephalopathy and died on day 14 of hospitalization.

## 3. Discussion

Stenosis or obstruction at the origin of the celiac artery is not uncommon and is reportedly found in 7.3% of cases on abdominal angiography.^[1]^ Causes include arteriosclerosis, median arch ligament, and aortitis.^[2]^ Although most patients are asymptomatic, MALS can cause aneurysms in the collateral blood vessels of the pancreaticoduodenal arcade or painful abdominal symptoms due to ischemia caused by stenosis of the celiac artery.

Biliary bleeding may be caused by trauma, vascular anomalies, malignancy, or stones and may be iatrogenic.^[3]^ In recent years, with the development of hepatobiliary procedures, iatrogenic biliary bleeding has been reported in patients undergoing percutaneous transhepatic biliary drainage or endoscopic drainage.^[4]^ Biliary bleeding after CMS placement is thought to result from compression or injury of the arterial wall by stent placement, biliary infection, or direct arterial invasion by a malignant tumor.^[5]^ Anatomically, the common bile duct is often penetrated by the right hepatic artery or pancreatoduodenal artery. In the present case, there was no aneurysm on angiography but bleeding was observed from the irregular vessels associated with tumor invasion, suggesting that the PSPDA associated with MALS was compressed by placement of the CMS and disrupted by direct invasion of the PC, resulting in massive biliary bleeding.

Symptomatic MALS requires radical treatment with resection of the median arcuate ligament, which has been reported to be useful for improving hepatic blood flow during pancreaticoduodenectomy and protecting against aneurysms. TAE has been reported to be useful for asymptomatic patients with aneurysms.^[6]^

Biliary bleeding is often not identified by upper gastrointestinal endoscopy, and contrast-enhanced CT or angiography is useful for diagnosis. When identified, the bleeding point can be compressed directly by CMS with a hemostatic effect.^[7]^ Therefore, cases of biliary bleeding during stenting are very rare.^[8]^ Yamaura et al reported that tumor shrinkage in response to chemotherapy can lead to stent displacement, causing bleeding by release of the pressure exerted by the stent and mechanical stimulation during displacement.^[9]^

In recent years, TAE has become the standard treatment for hemorrhage in the biliopancreatic area because it is minimally invasive and useful. However, surgical treatment such as aneurysmectomy, ligation of the incoming artery, and revascularization are selected in cases where the arteries in the pancreaticoduodenal area have complex branches and shapes that may make it difficult to approach the target vessel and increase the risk of extensive organ ischemia.^[10]^

Angiography is often the first choice for unstable vitals. In the present case, we performed resuscitative endovascular balloon occlusion of the aorta in the intensive care unit and the patient’s hemodynamics improved. However, transfer to a CT room is difficult. We performed emergency upper gastrointestinal endoscopy after hematemesis, and biliary bleeding was confirmed, placing a CMS in the bile duct to achieve temporary hemostasis. Subsequent contrast-enhanced CT confirmed bleeding from the PSPDA, and hemostasis was achieved using TAE. Chemotherapy had just been started, so the stent was not dislodged in response to chemotherapy, but rather because of bleeding from the PSPDA that developed from the superior mesenteric artery and led to sudden deterioration of the patient. We opted for a 10-mm CMS considering the diameter of the bile duct, and we believe it was an appropriate diameter because it was not overextended. However, although CMS has hemostatic effects, it has also been reported to exert pressure on the arterial wall and cause irritation. Therefore, plastic stents may be preferable for reducing mechanical stimulation.

MALS is not rare, and the number of cases of PC cases is expected to increase. Moreover, with advances in chemotherapy, these patients are expected to live longer but experience more complications. Preoperative CT should be performed to fully understand the location of the tumor, bile duct, and blood vessels when selecting a stent, and the possibility of fatal biliary hemorrhage should be considered.

## Author contributions

**Writing – original draft:** Ryosuke Nakatsubo

**Writing – review & editing:** Atsushi Sofuni, Takayoshi Tsuchiya, Kentaro Ishii, Reina Tanaka, Ryosuke Tonozuka, Shuntaro Mukai, Kazumasa Nagai, Yukitoshi Matsunami, Kenjiro Yamamoto, Hiroyuki Kojima, Hirohito Minami, Noriyuki Hirakawa, Kyoko Asano, Takao Itoi.
